# Probing the Mechanism of Action of Cry41Aa on HepG2 through the Establishment of a Resistant Subline

**DOI:** 10.3390/toxins14050319

**Published:** 2022-04-29

**Authors:** Wided Souissi, Tweedie Alistair, Barbara Domanska, Eva Fortea, Michelle J. West, Jean-Louis Schwartz, Neil Crickmore

**Affiliations:** 1 School of Life Sciences, University of Sussex, Brighton BN1 9QG, UK; A.Tweedie@sussex.ac.uk (T.A.); domanskab7@gmail.com (B.D.); M.J.West@sussex.ac.uk (M.J.W.); N.Crickmore@sussex.ac.uk (N.C.); 2 Departement of Pharmacology et Physiology, University of Montreal, Montreal, QC H3C 3J7, Canada; fortea.eva@gmail.com (E.F.); Jean-louis.Schwartz@umontreal.ca (J.-L.S.)

**Keywords:** cry toxin, parasporin, HepG2, AQP9

## Abstract

Cry41Aa, also called parasporin-3, belongs to a group of toxins from the entomopathogenic bacterium *Bacillus thuringiensis* that show activity against human cancer cells. Cry41Aa exhibits preferential cytocidal activity towards HL-60 (human promyelocytic leukaemia cells) and HepG2 (human liver cancer cells) cell lines after being proteolytically activated. To better understand the mechanism of action of Cry41Aa, we evolved resistance in HepG2 cells through repeated exposure to increasing doses of the toxin. Concentrations of Cry41Aa that killed over 50% of the parental HepG2 cells had no significant effect on the viability of the resistant cells and did not induce either pore formation or p38 phosphorylation (both characteristic features of pore-forming toxins). Preliminary RNA sequencing data identified AQP9 as a potential mediator of resistance, but extensive investigations failed to show a causal link and did not support an enhanced cell repair process as the resistance mechanism.

## 1. Introduction

The development of resistance to treatments used to counter diseases is a major obstacle. This phenomenon was observed in insect models that developed resistance to *Bacillus thuringiensis* (Bt)-based biopesticides as well as in cancer patients who developed resistance to chemotherapeutic agents. Understanding the basis of the resistance mechanisms is important for developing management strategies.

In insect models, the proposed mechanisms of resistance were associated with alteration in any step in the mechanism of action of Bt Cry toxins. Observed mechanisms of Cry toxin resistance include defects in receptor binding [1], altered activation of Cry toxins by midgut proteases [2], elevated immune response [3], and enhanced esterase production [4].

On the other hand, the principal mechanisms that are associated with the resistance of cancer cells to chemotherapy treatment include altered membrane transport involving the P-glycoprotein product of the multidrug resistance (MDR) gene as well as other associated proteins, altered target enzyme (e.g., mutated topoisomerase II), decreased drug activation, increased drug degradation due to altered expression of drug-metabolising enzymes, drug inactivation due to conjugation with glutathione, subcellular redistribution, drug interaction, enhanced DNA repair, and apoptosis failure as a result of mutated cell cycle proteins such as p53 [5].

One of the main approaches that has been utilised to study the resistance mechanisms in insects and cancer cell lines is to evolve a resistant population/subline to the toxic agent. The molecular alterations associated with resistance can then help in the clarification of the mechanisms of this change in phenotype.

In this study, a HepG2 cell line resistant to activated Cry41Aa was generated. Cellular and molecular changes were investigated in order to help unravel the mechanism of action of Cry41Aa.

## 2. Results

### 2.1. Generation and Characterisation of a Resistant HepG2 Cell Line

We established a resistance profile in HepG2 cells by culturing them in stepwise increasing concentrations of activated Cry41Aa. After 8 months of selection, the cells were able to tolerate a dose of 50 µg/mL.

**Figure 1:**
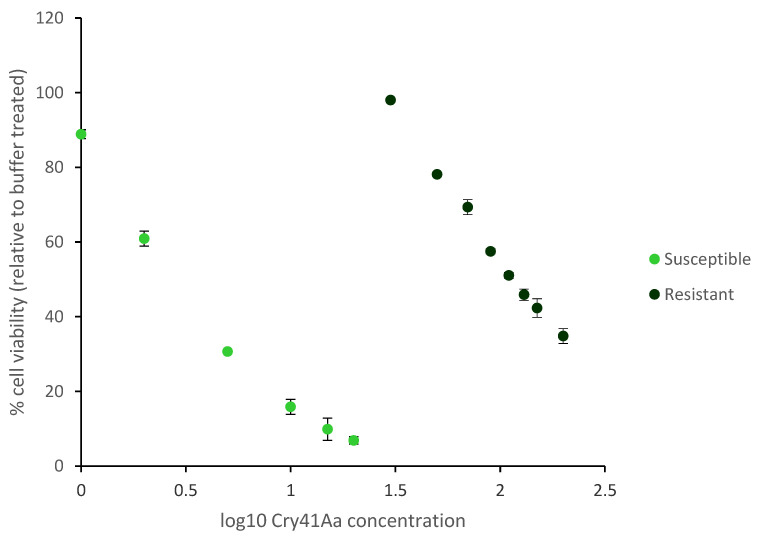
The establishment of a resistant HepG2 cell line. Cell viability was measured using CellTiter-Glo assay.

At the end of the selection process, we evaluated sensitivity to Cry41Aa of the evolved (HepG2R) and the parental cell lines. A considerable difference in the metabolic capacity of both cell lines post Cry41Aa treatment was shown. ATP levels, an indicator of cell viability, were shown to be significantly reduced in susceptible but not in resistant HepG2R. Using Statistical Package for the Social Sciences software (SPSS), Probit Regression analysis, the EC_50_ values of the resistant and susceptible cells were then determined as 113.3 μg/mL and 2.76 μg/mL, respectively (Figure 1). The resistance index was therefore equal to 41, a value that is within the range seen when resistant cells to chemotherapeutic agents were selected [6,7].

**Figure 2:**
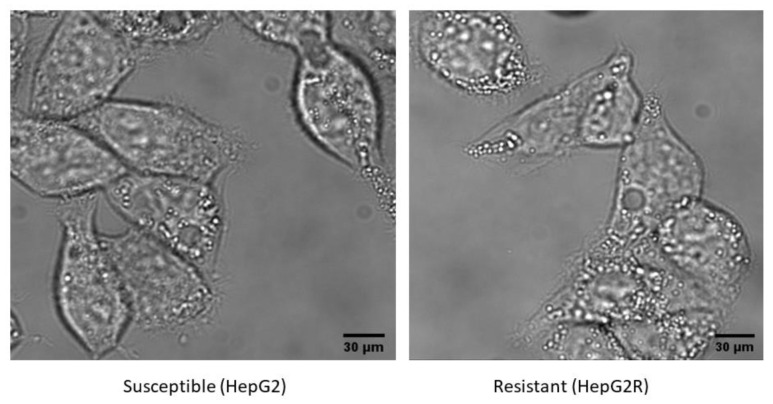
Morphological analysis of susceptible and resistant HepG2 cell lines.

Morphological analysis showed no obvious alterations between the two cell lines (Figure 2).

**Figure 3:**
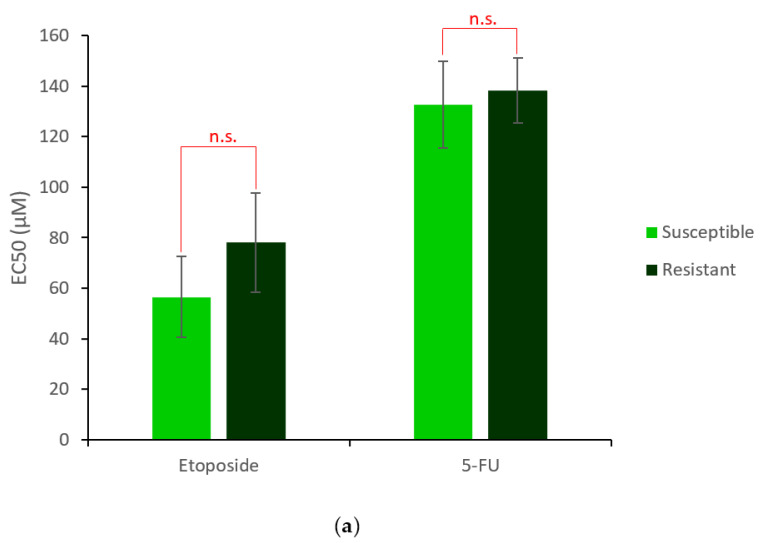

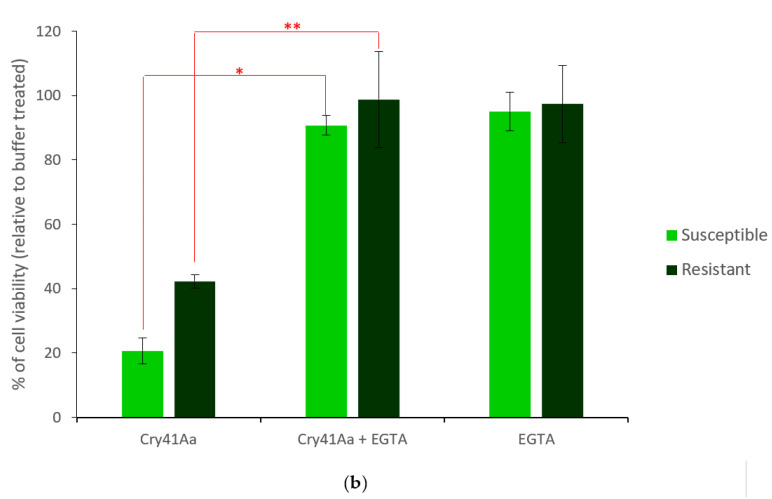
Assessment of the response of HepG2 cell lines to chemotherapeutic drugs and to Cry41Aa in the presence or absence of EGTA. (**a**) Effect of Etoposide and 5-FU n.s. = non-significant difference. (**b**) Effect of EGTA on Cry41Aa toxicity. * *p* = 0.0005, ** *p* = 0.0001.

Knowing the chemo-sensitivity of HepG2 cells towards Etoposide and 5-Fluorouracil (5-FU) [8,9], HepG2R cells were exposed to these two drugs to investigate the possible development of cross resistance. Our results showed no significant differences in the cytotoxic effect of either drug between the two cell lines (Figure 3a).

A detailed study on the effect of different metal ion chelators on Cry41Aa activity was carried out by Domanska et al. [10], who showed that ethylene glycol bis(β-aminoethyl ether)-N,N,N’,N’-tetraacetic acid (EGTA) inhibited the effect of Cry41Aa on susceptible HepG2. We confirmed this finding and further showed that this chelator also caused a significant reduction in the cytotoxicity of Cry41Aa against resistant HepG2 cells (Figure 3b).

### 2.2. Characterisation of HepG2R Cell Response to Cry41Aa Compared with the Parental Cell Line

**Figure 4:**
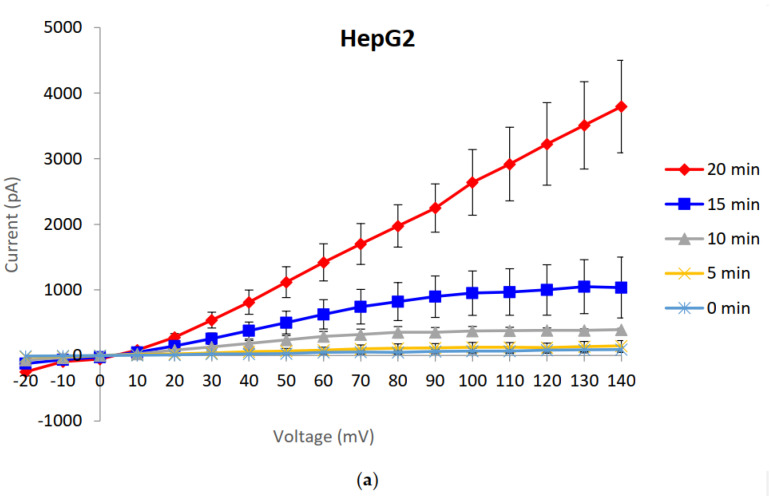

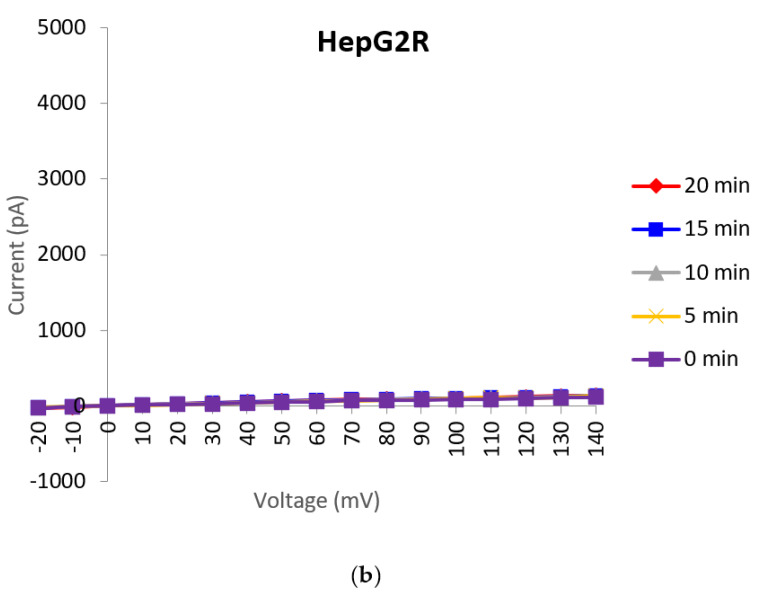
Whole-cell patch clamp recordings from a time course experiment of HepG2 and HepG2R cells exposed to activated Cry41Aa. (**a**) Whole-cell patch clamp recordings in HepG2. (**b**) Whole-cell patch clamp recordings in HepG2R. (**a**,**b**) Error bars indicate the standard error of the mean. The lines show the mean currents from three representative experiments from three different patched cells.

Macroscopic current activities were assessed using whole-cell patch clamping. The recordings showed the induction of large currents in susceptible cells (Figure 4a) unlike in resistant HepG2R that were exposed to a same dose of Cry41Aa (Figure 4b).

**Figure 5:**
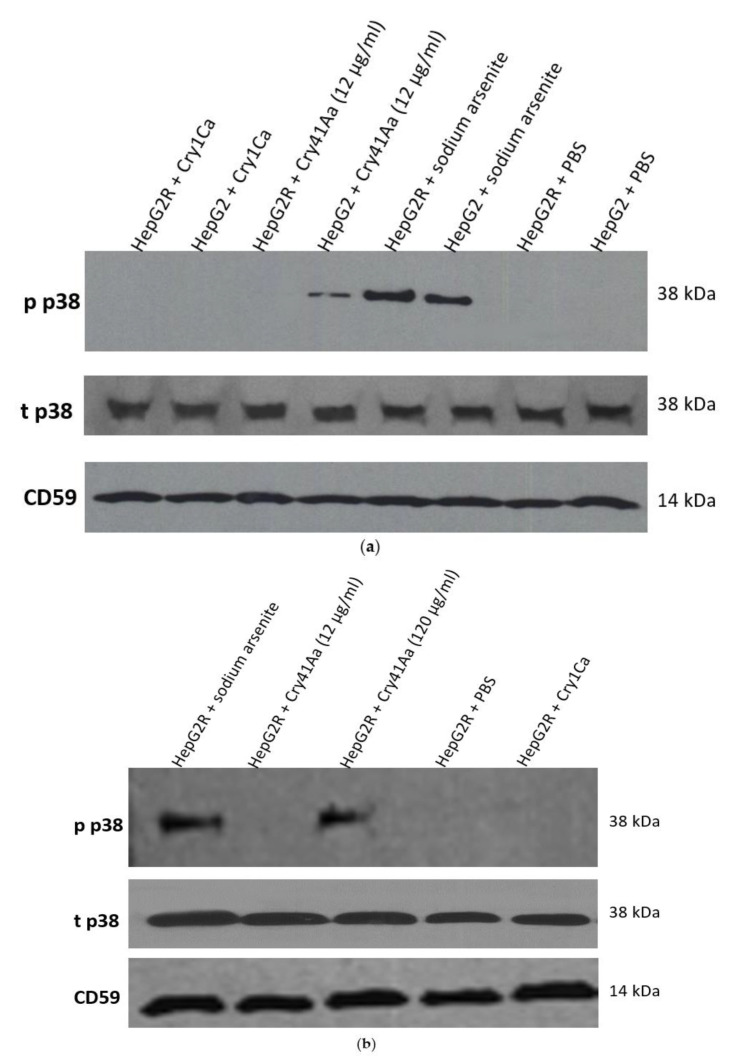
Characterisation of human cancer cell response to Cry41Aa. (**a**) Effect of Cry41Aa on total (t p38) and phosphorylated (p p38) p38 levels in HepG2 and HepG2R cells. (**b**) Effect of Cry41Aa concentration on total (t p38) and phosphorylated (p p38) p38 levels in HepG2R cells. (**a**,**b**) CD59 was used as a loading control.

It was previously demonstrated that the osmotic stress produced after the formation of pores by different pore-forming toxins (PFT) in target cells induces a MAPK p38-phosphorylation-response that is crucial to prevent bacterial infection [11]. Previous studies have shown that phosphorylation of p38, a mitogen-activated protein kinase (MAPK), was activated in HepG2 cells exposed to Cry41Aa [10]. Because this cellular defence was shown to be conserved in mammalian cells attacked by PFTs [12], it was interesting to investigate if this response was similar in the HepG2R cell line. P38 phosphorylation was observed in the case of HepG2 cells exposed to 12 µg/mL of Cry41Aa (Figure 5a), but only when this dose was increased to 120 µg/mL was p38 phosphorylation observed in HepG2R cells (Figure 5b). 

We then moved on to investigate the morphological changes in these cell lines upon Cry41Aa treatment. HepG2 and HepG2R cells were treated with Cry41Aa, then cell morphology was visualised and monitored over time. Following exposure to 2.5 µg/mL of Cry41Aa, HepG2 cells swelled, burst, and died; on the other hand, this dose did not have any obvious effect on HepG2R. However, swelling followed by recovery was observed in the case where HepG2R cells were exposed to 60 µg/mL (Supplementary Video S1) or when HepG2 cells were exposed to 1 µg/mL (Supplementary Video S2).

Cell swelling occurs when the cell loses its ability to control the movement of ions and water in and out, indicating osmotic imbalance [13]. Colloid osmotic lysis is the most accepted model of Cry toxin mode of action [14]. In addition, water influx was previously suggested to directly determine necrotic cell death induced by the *Bacillus thuringiensis* Cry toxin via aquaporins that are water-selective membrane channels and shown biophysically to conduct water, glycerol, and a broad range of non-charged solutes [15].

### 2.3. Differential Expression of AQP9 in the Resistant Cell Line

Preliminary RNA sequencing data of both susceptible and resistant HepG2 cell lines identified aquaporin 9 (AQP9) as being differentially expressed which, due to the involvement of aquaporins in the mechanism of action of an insecticidal Cry protein as discussed above, might represent a potential candidate for a resistance mechanism [16]. We therefore investigated the possibility that increased expression of AQP9 in HepG2R could protect the cells from low doses of Cry41Aa, even though previous data indicated that increased expression of aquaporins facilitated the toxic process [15].

#### 2.3.1. Validation of AQP9 Expression Levels

**Figure 6:**
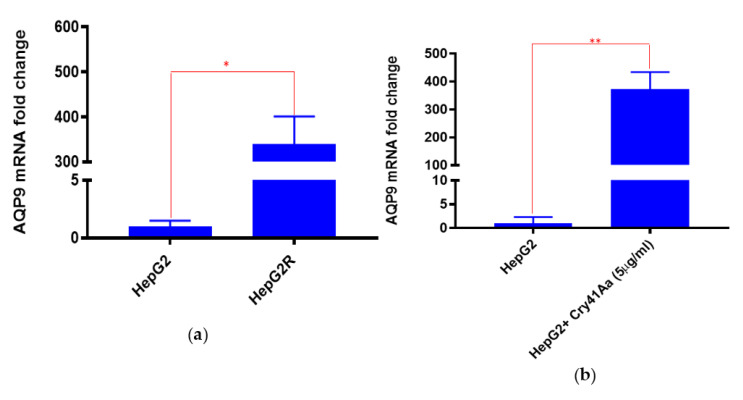
Validation of AQP9 mRNA levels in HepG2 and HepG2R. (**a**) AQP9 mRNA levels in HepG2 and HepG2R. (**b**) Effect of Cry41Aa on AQP9 mRNA levels. (**a**,**b**) Gene expression was analysed using the relative quantification (RQ) method. GAPDH was used as housekeeping gene. Error bars: RQ min/max. * *p* = 1.71 × 10^−6^; ** *p* = 3.85 × 10^−5^.

The gene expression levels of *aqp9* were initially evaluated in both parental and HepG2R cell lines. Our results showed that mRNA level of *aqp9* was significantly higher in resistant than in susceptible cells (around 100-fold difference) (Figure 6a). We hypothesised that Cry41Aa induces AQP9 expression, and that this upregulation may have a role in maintaining homeostasis and that in the resistant cell line, it is constitutively upregulated. We then investigated the expression level of *aqp9* in HepG2 pre- and post-treatment with 5 µg/mL of Cry41Aa for 30min (Figure 6b). AQP9 mRNA levels increased considerably (around 400-fold) in HepG2 cells upon Cry41Aa treatment, indicating that Cry41Aa induced AQP9 expression. As a result of this finding, we proposed a model for the role of AQP9 in the mechanism of action of Cry41Aa on HepG2. The model suggests that exposure of HepG2 to Cry41Aa leads to pore formation in the cell membrane through which water and other solutes will enter, causing osmotic imbalance. AQP9, induced by this action, will try to retain the osmotic balance via the transport of water, along with a number of other solutes, out of the cell. However, if there is insufficient AQP9, the effect of Cry41Aa is too great, causing cell swelling and leading to cell lysis and death. In HepG2R, the high expression of AQP9 could play a role in quickly reducing the osmotic gradient and restoring the cell to a healthy state, preventing lysis.

Based on the proposed model, we tested whether a transient increase in AQP9 in HepG2 could result in the acquisition of immunity to Cry41Aa. This transient increase in AQP9 expression could be achieved by exposure of HepG2 to Cry41Aa based on the fact that Cry41Aa was shown to induce AQP9 expression (Figure 6b). However, since the 5 µg/mL used in Figure 6b would eventually kill most of the cells, we decided to work with a sub-EC50 dose that could result in the increase in AQP9 levels and in the meantime preserve cell viability.

**Figure 7:**
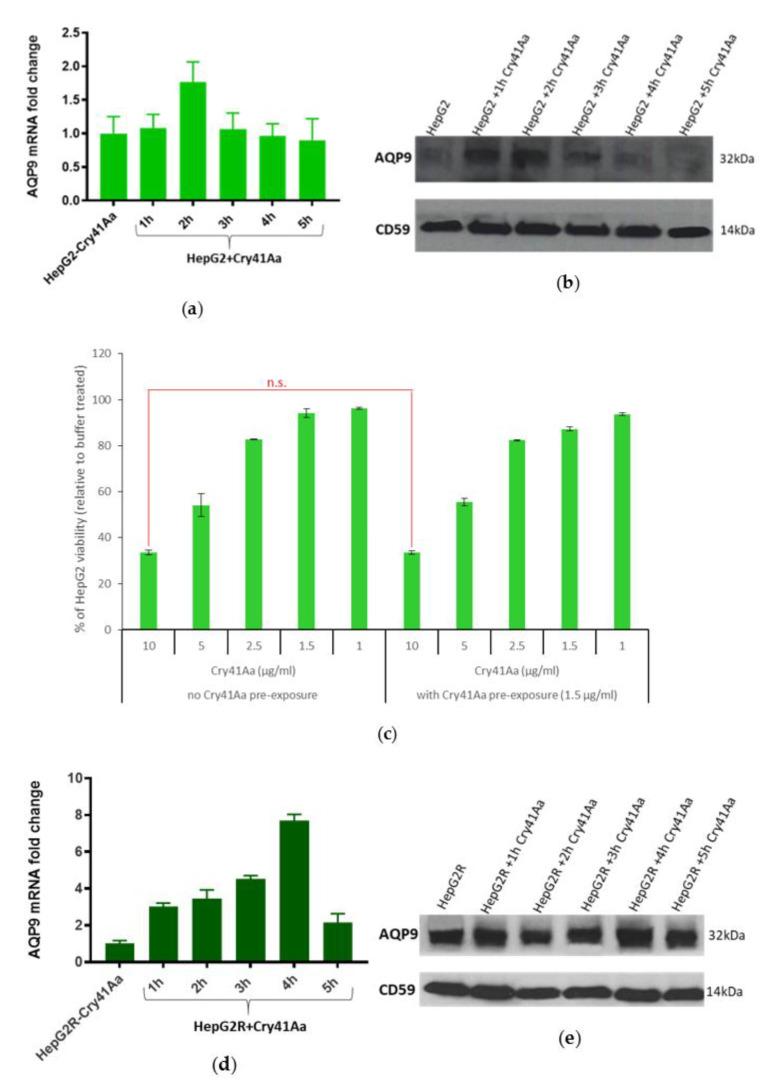

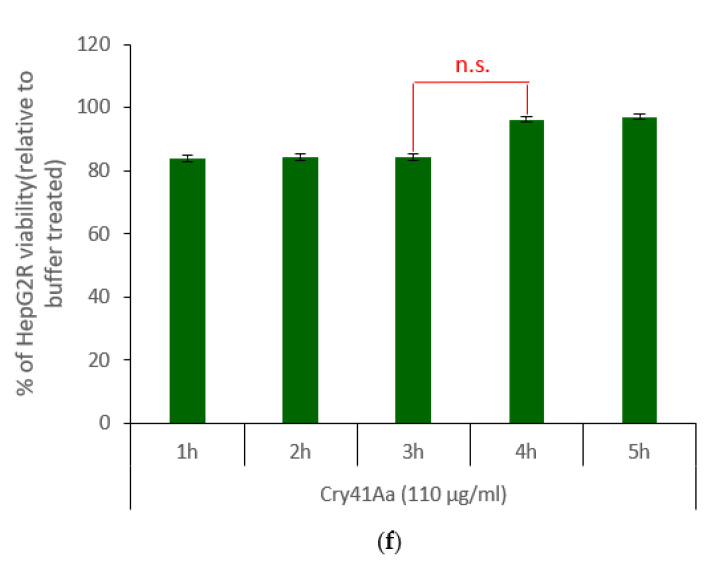
Effect of Cry41Aa on AQP9 expression. (**a**,**d**) AQP9 mRNA levels in HepG2 and HepG2R following exposure to Cry41Aa over time. Gene expression was analysed using the relative quantification (RQ) method. Error bars: RQ min/max. (**b**,**e**) Effect of Cry41Aa on AQP9 expression over time. CD59 was used as the loading control. (**c**) Effect of pre-exposure to Cry41Aa on HepG2 susceptibility. n.s. = non-significant difference. (**f**) Susceptibility of HepG2R cells to Cry41Aa measured using CellTiter-Glo assay. n.s. = non-significant difference.

Therefore, we assessed the effect of 1.5 µg/mL Cry41Aa on AQP9 expression over time. HepG2 cells were exposed to this dose of Cry41Aa, and mRNA and protein levels were analysed at different time points (Figure 7a,b). After 2 h of exposure to Cry41Aa, AQP9 mRNA and protein levels reached their maximal levels, then dropped gradually to reach a similar level to that of cells treated with buffer only.

Since incubation with Cry41Aa for two hours increased AQP9 protein expression, based on our hypothesis that AQP9 has a role in cell defence, HepG2 cells were incubated with Cry41Aa (1.5 µg/mL) for 2 h. The medium was then removed, and the cells were washed with PBS followed by the addition of different concentrations of Cry41Aa. Cell viability was evaluated in order to see whether the cells had acquired immunity to Cry41Aa due to AQP9 expression, which would result in a decrease in the susceptibility level (Figure 7c). According to the cell viability assay, the sensitivity to Cry41Aa was similar between the cells pre-exposed to the sub-EC50 dose of Cry41Aa and the cells that were not pre-exposed to Cry41Aa, indicating that AQP9 levels do not correlate with Cry41Aa susceptibility.

The same process was followed for the HepG2R cell line in order to establish any relationship between AQP9 and resistance to Cry41Aa. Initially, the cells were exposed to 110 µg/mL of Cry41Aa. mRNA (Figure 7d) as well as protein levels (Figure 7e) of AQP9 were assessed at different time points. mRNA levels of AQP9 increased gradually over time following the exposure of cells to Cry41Aa. This level reached its maximum after 4 h, then decreased to reach approximately the same level as that of the cells treated with buffer only. An obvious increase in AQP9 protein levels in cells that were exposed to Cry41Aa over that 4 h period was also observed. The cytotoxic effect of Cry41Aa was assessed at these different time points (Figure 7f), showing that after 4 h of treatment, there was a small but insignificant increase in HepG2R viability.

#### 2.3.2. Correlation between AQP9 Expression and Cry41Aa Susceptibility

**Figure 8:**
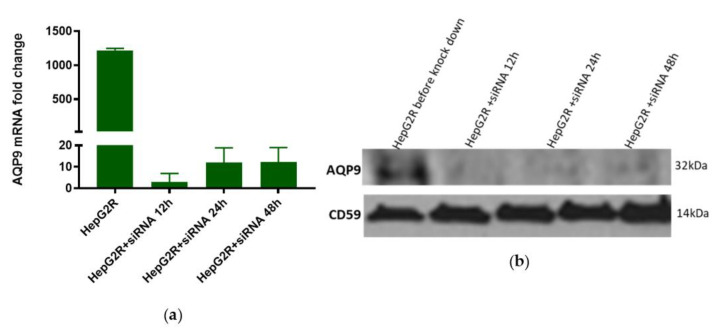

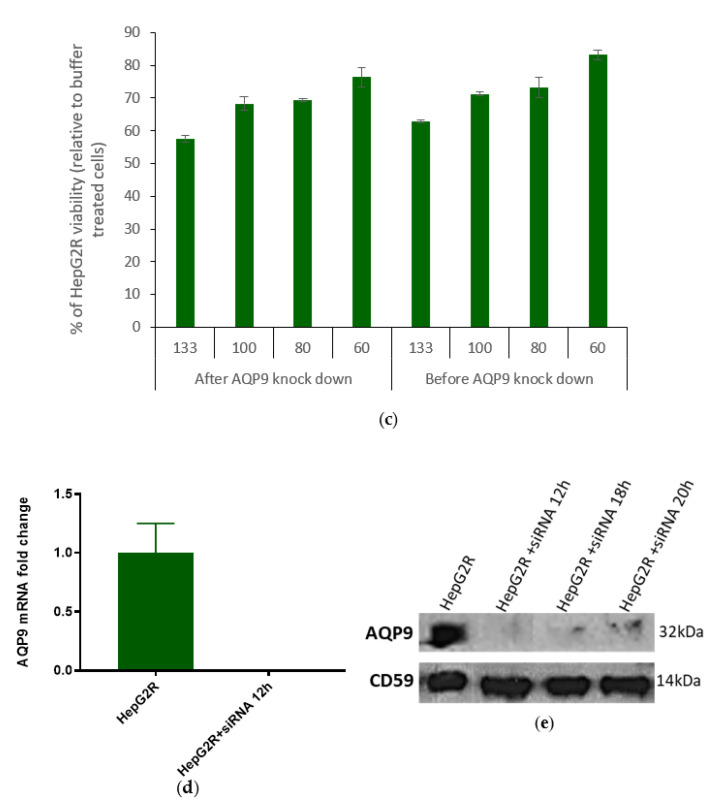
Effect of AQP9 knockdown on Cry41Aa susceptibility. (**a**,**d**) AQP9 mRNA levels in HepG2 and HepG2R following knock down. Gene expression was analysed using the relative quantification (RQ) method. GAPDH was used as a housekeeping gene. Error bars: RQ min/max. (**b**) AQP9 expressions levels in HepG2R following knockdown. (**c**) Effect of AQP9 knockdown in HepG2R on their susceptibility to Cry41Aa, measured using CellTiter-Blue assay. (**e**) Effect of Cry41Aa addition on AQP9 expression in HepG2R after knockdown.

Knockdown of AQP9 in HepG2R was then performed in a further attempt to investigate its possible role in the resistance mechanism. Our results showed successful silencing at mRNA (Figure 8a) as well as at protein levels (Figure 8b). AQP9 knockdown was optimal when cells were transfected with siRNA for 12 h.

Susceptibility of the transfected HepG2R cells to Cry41Aa was then assessed in order to confirm the role, if any, of AQP9 in resistance. The cells were initially transfected with siRNA for 12 h, Cry41Aa was added at different concentrations, and cell viability was evaluated (Figure 8c). The same level of toxicity of activated Cry41Aa against transfected and non-transfected cells was observed, indicating that the knockdown of AQP9 in the HepG2R cell line did not affect its susceptibility to Cry41Aa.

In parallel to the cell assay, the level of expression of AQP9 was monitored at each step. AQP9 mRNA was not detected in transfected cells (Figure 8d). At the protein level, a large decrease in the level of AQP9 was observed (Figure 8e). This signal slightly increased after incubation of the cells with Cry41Aa for a further 6 h (HepG2R + siRNA 18h) and at the time point when cell viability was measured (HepG2R + siRNA 20h), although it remained much lower than pre-knockdown levels.

## 3. Discussion

Drug-resistant cell line models have previously been used to unravel the resistance mechanism, leading to a deeper understanding of the toxic agent’s mode of action. In our study, the method utilised to generate a HepG2 cell line resistant to Cry41Aa was continuous exposure of cells to increasing doses of Cry41Aa over time. The established subline was 41 times more resistant than the parental line. This method, as well as the pulse treatment strategy, were previously employed and led to the successful establishment of HepG2 cell lines resistant to particular chemotherapeutic drug(s) [17,18,19]. In insect models, the selection of resistant strains was conducted by incorporating toxins into the diet at progressively increasing concentrations [20].

Morphological changes were observed in human cancer cells treated with Cry41Aa, which seemed to depend on their degree of susceptibility as well as Cry41Aa concentration. Microscopic observations of HepG2 and HepG2R cell lines exposed to EC50 doses of Cry41Aa showed cell swelling that progressed to lysis and death. On the other hand, when exposed to sub-EC50 doses of Cry41Aa, initial cell swelling was observed, followed by recovery and survival. This ability to overcome Cry41Aa damage could be correlated with lower expression of Cry41Aa cell surface receptor or a more efficient pore repair mechanism.

Previous studies have shown that two mitogen-activated protein kinase (MAPK) pathways were up-regulated in *Caenorhabditis elegans* in response to Cry5B toxin. Both of these MAPK pathways provided a significant cellular defence against the toxin, and this defence was shown to be conserved in mammalian cells attacked by PFTs [12]. Osmotic stress produced after the formation of pores by different PFTs in the target cells was shown to induce a MAPK p38 phosphorylation response [11]. Previous studies have demonstrated that toxins where mutations were made in regions essential for pore formation activity were unable to induce the p38 response. Following treatment of HaCaT cells with a low concentration (<10 ng/mL) of α-toxin, phosphorylation of MAPK p38 was activated. The pore formation activity of the toxin was correlated with activation of p38 since a pore formation mutant was unable to induce this activation [21].

MAPK signalling could trigger cellular response following membrane pore formation. Our previous data suggested that Cry41Aa causes small membrane injuries with an estimated pore diameter <1 nm, close to values recorded for insecticidal Cry toxins [22,23]. In general, cellular repair responses to small injuries (nm range) caused by PFTs rely on pore removal rather than membrane patching by Ca^2+^-dependent exocytosis. Such mechanisms, which could be a downstream effect of the MAPK signalling, might include endosomal degradative pathways and exosomal shedding [24]. These two processes were observed for a different Cry toxin, Cry5B, following in vivo infection of *C. elegans* [25]. In addition, the recovery process of human cancer cells in response to pore-forming toxins was previously observed to involve microvesicle shedding, where a toxin is sequestered into domains in the plasmalemma that bleb outwards and bud from the cell surface into the medium [26,27].

Preliminary RNA sequencing of HepG2 and HepG2R cell lines had identified AQP9, being differentially expressed, as a potential candidate since this membrane channel has been implicated in the mechanism of action of Cry1Aa against Sf9 cells expressing a Bt receptor [15]. AQP9 mRNA as well as protein levels were shown to be significantly higher in the resistant compared with the susceptible cell line. Cry41Aa was also shown to induce AQP9 expression. This led us to produce a model of action for Cry41Aa where AQP9 was suggested to be involved in the response process of intoxicated cells through regulation of the osmotic imbalance and maintaining their homeostasis. However, AQP9 knockdown did not have any effect on Cry41Aa activity, suggesting that there was no correlation between AQP9 expression and susceptibility to Cry41Aa, at least in the resistant subline.

To conclude, we can provide no evidence for resistance being associated with an enhanced repair mechanism, whether involving aquaporins or not. More likely is that resistant/less susceptible cell lines have less receptor expression and thus need higher doses of Cry41Aa to elicit a toxic effect. Although the patch clamping experiments were performed on whole cells, which could therefore be capable of mounting a cell repair process, the observation that no effect was observed in the resistant line soon after adding the toxin (5–10 min) further supports the idea that resistance is due to the lack of pore formation rather than the induction of a repair mechanism.

## 4. Materials and Methods

### 4.1. Crystal Protein Harvesting and Activation

Bt transformants were grown in LB agar plates supplemented with 5 μg/mL chloramphenicol for 3 days at 30 °C. Sporulation and production of crystals were monitored using a phase contrast microscope. Sporulated cells containing crystals were scraped off the plates and sonicated. Crystal proteins were then solubilised in 50 mM sodium carbonate at pH 10.5 in the presence of 5 mM Dithiothreiotol (DTT) at 37 °C for 1 h. Supernatant was collected and treated with chymotrypsin (1 mg/mL) at 37 °C for 1 h. Complete mini EDTA-free protease inhibitor (Roche) was finally added to the activated samples to prevent further proteolysis.

### 4.2. Protein Purification

Purification of activated Cry41Aa was carried out using a 1 mL Resource Q column (GE Healthcare Life Sciences) connected to an ÄKTA Purifier-FPLC System. Samples were injected in 10 mM CAPS (pH 10.5) and a linear increase in the gradient of NaCl (0 to 1 M) was applied at a flow rate of 1 mL/min for 25 min. Protein concentration was determined by the Bradford method with a Bio-Rad Protein Assay Kit using bovine serum albumin as the standard.

### 4.3. Cell Cultures

HepG2 cell line was purchased from the European Collection of Cell Cultures (ECCAC; Salisbury, UK). HepG2R, the Cry41Aa-resistant subline of HepG2, was developed in the lab. Cells were cultured in Dulbecco’s Modified Eagle’s Medium (DMEM, Gibco) or Roswell Park Memorial Institute (RPMI) 1640 Medium provided with 1% penicillin-streptomycin–neomycin (PSN) antibiotic mixture and 10% foetal bovine serum (FBS) in a humidified atmosphere containing 5% CO_2_ at 37 °C. The cells were split when they reached 70–80% confluence.

### 4.4. Development of the Resistant HepG2R Cell Line

HepG2 cells were cultured in stepwise increasing concentrations of purified chymotrypsin-activated Cry41Aa. Initially, the cells were seeded at around 20% confluence and Cry41Aa treatment was carried out after 24 h, with a commencing dose of 0.1 μg/mL. As the cells became confluent, they were sub-cultured in the usual manner, and the increase in Cry41Aa dose generally followed the pattern of doubling the concentration unless the cells appeared to have not tolerated the previous treatment, in which case, they were allowed to recover in toxin-free medium and exposed to a less concentrated dose. A range of different concentrations was used (0.1; 0.2; 0.3; 0.4; 0.8; 1.6; 2.6; 4; 8; 10; 15; 20; 30; 50 μg/mL) over a period of 8 months.

### 4.5. Cell Assays

Assays were performed in 96-well plates (Nunc). Each well received 90 μL of cell suspension at a density of 22,500 cells per well and cultured overnight (at 37 °C/5% CO_2_ humidified air) before 10 μL of the test sample was added. The experiments were set up in triplicate. The mock control wells received 90 μL of cell suspension and 10 μL of the appropriate buffer. The wells that contained 100 µL of cell culture medium served as background fluorescent/luminescent controls. The readings were carried out 24 h following treatment using GloMax-Multi Detection System (Promega) according to the assay(s) instructions. The fluorescent (CellTiter-Blue) or luminescent (CellTiter-Glo) signal in the background control wells was subtracted from each experimental value.

To assess cell response to Cry41Aa in the presence of EGTA, cells were pre-exposed to 5 mM of EGTA or water (mock) for 30 min, followed by the addition of Cry41Aa (12 μg/mL for HepG2 and 120 μg/mL for HepG2R). Viability readings were taken 6 h after Cry41Aa addition using CellTiter-Blue assay. Student’s t-test was used to determine if there is a significant difference between the means of two groups, with a *p*-value of less than 0.05 (*p* ≤ 0.05) being statistically significant.

### 4.6. Microscopy

HepG2 and HepG2R cells were seeded at a density of 4 × 10^4^ cells/mL in a microscope chamber slide. The next day, pictures were taken using Zeiss Axiovert 200M, 63x DIC objective. Images were analysed using Image J software.

### 4.7. Western Blots

For the p38 MAPK experiments, HepG2 and HepG2R cells were treated with purified chymotrypsin-activated Cry41Aa for 30 min before being lysed in RIPA buffer. Sodium arsenite (0.5 mM) was used as a positive control since it is a potent p38 inducer [28]. On the other hand, trypsin-activated Cry1Ca was used as a negative control because this insecticidal toxin was expressed in the same acrystalliferous Bt strain as recombinant Cry41Aa and shares a similar three-domain Cry toxin fold but has no known cytotoxic effect on human cells. For AQP9 knockdown, HepG2R cells were seeded at a density of 8 × 10^4^ cells/mL, and the transfection solution was added. At different time points, non-transfected and transfected HepG2R were either lysed or further incubated with Cry41Aa at a concentration of 133 µg/mL for 6 h, then lysed using RIPA, and the concentrations of extracted proteins were measured using the Bradford method (Bio-Rad Protein Assay Kit) with BSA as the standard.

Initially, 15 and 20 µg of proteins per well for p38 and AQP9, respectively, were run on SDS-PAGE gels (7.5–12%). Using a Bio-Rad Trans-Blot Semi-Dry Transfer Cell system (100 mA for 30–75 min), they were transferred onto a nitrocellulose membrane (Bio-Rad, 0.45 µm). The membrane was then incubated with blocking solution (PBS containing 0.02% Tween-20 (PBST) and 3% BSA or 5% non-fat dry milk) and later incubated with the appropriate primary antibody (tp38 (Cell Signaling Technology, Leiden, Netherlands, 9101S); pp38 (Thermo Fisher Scientific, Leicestershire, UK, (MA5 15182); CD59 (abcam, Cambridge, UK, ab126777); AQP9 (Thermo Fisher scientific, Leicestershire, UK, PA5-51285)) at 4 °C overnight with shaking.

The next day, the membrane was washed with PBST, then incubated for 1 h at room temperature with secondary HRP-conjugated antibody (abcam, ab97051). After a final wash, the membrane was incubated in chemiluminescent detection solution and finally either exposed to X-ray film (FUJI medical X-ray film) or placed in UVP ChemStudio imaging system (analytikjena).

### 4.8. Whole-Cell Patch-Clamp

HepG2 cells were seeded at 5 × 10^4^ cells/mL on 24 mm circular glass coverslips 24–48 h before experiments. Cells were washed three times with the bath solution (140 mM NaCl, 5 mM KCl, 1.1 mM MgCl_2_, 1.1 mM CaCl_2_, 10 mM HEPES, pH 7.4) and mounted inside a coverslip holder fitted to the microscope stage (IMT-2, Olympus). Then, 1 mL of the bath solution was added. Soda lime pipettes (Kimble Chase) were filled with the pipette solution (140 mM KCl, 1.1 mM MgCl_2_, 0.1 mM EGTA, 10 mM HEPES, pH 7.4) and had a resistance of around 4 MΩ. The resistance of the seals was in the range of 2–10 GΩ. A −20 mV holding potential and trains of 17 command voltage steps (1 s duration, ranging from −20 to 140 mV, in increments of 10 mV) were applied before and 20 min after the addition of Cry41Aa (12 μg/mL) to the bath. The command voltages were generated and the whole-cell currents were recorded and processed with an Axon Digidata 1550 converter (Molecular Devices) and Axopatch—1D patch-clamp amplifier (Molecular Devices). Currents were filtered at 10 kHz and sampled at 50 kHz. Data collection and analysis were conducted using the pCLAMP 10.6 software (Molecular Devices). The current–voltage (I/V) curves show the mean currents from three representative experiments on three different patched cells. Experiments were performed at room temperature.

### 4.9. RNA Extraction

RNA extraction was performed using QIAGEN RNeasy Plus Mini Kit according to the manufacturer’s instructions. The eluted RNA samples were stored at −80 °C until use.

### 4.10. Determination of RNA Concentration and RNA Integrity

RNA concentrations as well as their integrities were determined using the Agilent Bioanalyser 2100 as per the manufacturer’s instructions using an RNA 6000 Nano Kit. To 65 μL of filtered gel, 1 μL of RNA 6000 Nano dye concentrate was added. Then, 9 μL of this gel–dye mix was loaded into each of the marked wells on an RNA Nanochip placed on the chip priming station. After loading 5 μL of the RNA 6000 Nano Marker in each of the appropriate wells, 1 μL of each of the ladder and the sample were then loaded into the chip, which was later inserted in the Agilent 2100 Bioanalyser.

### 4.11. RNA-Seq and Analysis

RNA-Seq was performed by a third party. GATC Biotech undertook the RNA-Seq (INVIEWTM Transcriptome) of the purified RNA using Illumina sequencing. The generated FASTQ files were next analysed using Galaxy software (usegalaxy.org accessed date 20 May 2018).

### 4.12. cDNA Synthesis

First, 2 μg of total RNA was reverse transcribed to cDNA using High-Capacity cDNA Reverse Transcription Kit (Applied Biosciences Thermo Fisher Scientific, Leicestershire, UK, 4388950) according to the manufacturer’s instruction. Then, 20 μL of reaction was placed in a thermo-cycler that was set up as follows: 25 °C for 10 min followed by 37 °C for 120 min, then 85 °C for 5 min, and finally held at 4 °C.

### 4.13. qPCR

A 96-well plate was set up with a final reaction volume of 20 µL containing the following: 10 µL of Syber Green master mix (Applied Biosystems), 1 µL of cDNA, 1 µL of forward primer (200 mM), 1 µL of reverse primer (200 mM), and 7 µL of H_2_O. AQP9 was used as the target gene while GAPDH was used as the endogenous control. All reactions were performed in triplicate. RNAs that were subjected to cDNA synthesis without the reverse transcriptase were used as non-genomic DNA controls, while wells lacking cDNA served as negative controls. The qPCR was run on an Applied Biosystems StepOne™ Real-Time PCR System. Relative gene expression was calculated using the comparative cycle threshold (2^−ΔΔCT^) method. RQ estimates the difference at the level of gene expression against a calibrator (HepG2 drug sensitive line) (RQ of the calibrator = 1). The analysis was conducted employing the standard formula: RQ = 2^−ΔΔCt^ (where ΔΔCt = ΔCt for the sample (exp: HepG2 (2)) −ΔCt for the calibrator (HepG2)). GAPDH was used as a housekeeping gene.

The primers used in this experiment for AQP9 and GAPDH were:

AQP9 (FP: CTGGTGGAAAACTGCTGATCG; RP: CTGCAAATGCGTTCGCCAGAG) and GAPDH (FP: ATCCCTGAGCTGAACGGGAA; RP: GGCAGGTTTTTCTAGACGGC).

### 4.14. AQP9 Knockdown in HepG2R

AQP9 knockdown was achieved through the use of RNA interference (FlexiTube GeneSolution-QIAGEN, Manchester, UK). Initially, the transfection solution was prepared: 4 μL of Hyperfect, 1 μL of each siRNA (FlexiTube GeneSolution-QIAGEN) (giving a total concentration of 20 nM), and 95 μL OPT-MEM. The mixture was then vortexed and incubated for 5–10 min at room temperature to allow the formation of the transfection complexes. Next, 1 × 10^5^ cells per well were seeded in a 24-well plate to which the transfection solution was added. The plate was incubated in a humidified atmosphere containing 5% CO_2_ at 37 °C, and gene silencing was monitored using RT-qPCR and Western blot.

## Supplementary Materials

The following supporting information can be downloaded at: https://www.mdpi.com/article/10.3390/toxins14050319/s1, Video S1: HepG2R cell repair following Cry41Aa treatment; Video S2: HepG2 cell repair following Cry41Aa treatment.
